# Statistics on the Frequency of Neoplasms in the Dog

**Published:** 1896-07

**Authors:** 


STATISTICS ON THE FREQUENCY OF NEOPLASMS IN THE DOG. The number of dogs treated at the veterinary school in Berlin by Prof. Fröhner, as well as in the hospitals and in daily practice, reached a total of 60,471 for the period of 1886–94. From the number he removed 2871 tumors, about 5 per cent. of the general sickness. The exact nature of these tumors has only been determined in the cases treated at the veterinary school, for the diagnosis of neoplasms require, as a rule a microscopic examination, except for the fibromas, papillomas, lipomas, cysts, etc., whose histological characteristics are visible to the naked eye. Of the 643 tumors which were operated and analyzed, Fröhner gives the following statistics:

| | Per cent. of all tumors. | | Per cent. of all tumors. |
|---|---|---|---|
| 262 cancers | = 40 | 16 mucous cysts | = 2.5 |
| 97 fibromas | = 13 | 14 goitres | = 2.2 |
| 65 papillomas | = 10 | 9 ranulas | = 1.4 |
| 44 sarcomas | = 7 | 7 atheromas | = 1 |
| 39 lipomas | = 6 | 4 dermatoids | = 0.6 |
| 17 polyps of the vagina | = 2.6 | 2 angiomas | = 0.3 |

The same proportion held good in the 2228 tumors observed at the polyclinic. It follows that the frequency of cancer in the dog, 4 per cent. of all the tumors, is greater than was supposed.—Abstract of an article in the *Annales de Médecine Vétérinaire* (Jan., Feb., 1896), from the *Monatsheft für prak. Thierheilkunde*, B. vi. H. 1, 2, and 3.

